# Tango of dual nanoparticles: Interplays between exosomes and nanomedicine

**DOI:** 10.1002/btm2.10269

**Published:** 2021-11-24

**Authors:** Yabin Wang, Wenzhen Wang, Fangong Kong, Qiu Zhang, Jiaqi Xiao, Yi Zhang, Bing Yan

**Affiliations:** ^1^ State Key Laboratory of Biobased Material and Green Papermaking Qilu University of Technology, Shandong Academy of Science Jinan China; ^2^ Advanced Research Institute for Multidisciplinary Science Qilu University of Technology, Shandong Academy of Science Jinan China; ^3^ The Secondary Hospital, Cheeloo College of Medicine Shandong University Jinan China; ^4^ School of Environmental Science and Engineering Shandong University Qingdao China; ^5^ Rutgers Cancer Institute of New Jersey Rutgers State University of New Jersey New Brunswick New Jersey USA; ^6^ Institute of Environmental Research at Greater Bay Area, Key Laboratory for Water Quality and Conservation of the Pearl River Delta, Ministry of Education Guangzhou University Guangzhou China

**Keywords:** exosome, nanomedicine, nanoparticle, targeted drug delivery

## Abstract

Exosomes are lipid bilayer vesicles released from cells as a mechanism of intracellular communication. Containing information molecules of their parental cells and inclining to fuse with targeted cells, exosomes are valuable in disease diagnosis and drug delivery. The realization of their clinic applications still faces difficulties, such as lacking technologies for fast purification and functional reading. The advancement of nanotechnology in recent decades makes it promising to overcome these difficulties. In this article, we summarized recent progress in utilizing the physiochemical properties of nanoparticles (NPs) to enhance exosome purification and detection sensitivity or to derive novel technologies. We also discussed the valuable applications of exosomes in NPs‐based drug delivery. Till now most studies in these fields are still at the laboratory research stage. Translation of these bench works into clinic applications still has a long way to go.

Most eukaryotic cells secrete bilayer lipid vesicles named exosomes to maintain intracellular material hemostasis and intercellular communication. Exosomes originate from the endosomal compartments and are released from cells when fused with plasma membrane. Typically having a diameter of ~40–160 nm, exosomes are heterozygous in size and composition. The components, including information molecules such as DNAs, mRNAs, miRNAs, proteins, and lipids, are restrained inside or on the surface depending on their origin. Recent studies have shown that these information molecules retain the physiological status and functions of their parental cells.^1^, ^2^ Exosomes are ubiquitously found in almost all body fluids, such as blood, urine, saliva, breast milk, and cerebrospinal fluid, therefore they are valuable liquid biopsy biomarkers that are easily accessible for early disease diagnosis and prognosis.^3^, ^4^ Because of these reasons, exosomes are extensively studied in recent years for potential clinical applications.

As an important mechanism of intercellular communication, exosomes mediate material exchange among cells by fusing with recipient cells and transmit their cargos into them, a process leading to disease advancing or restraining.^5^, ^6^ Because of the homology, exosomes fuse with target cells in a high efficiency without causing undesirable side effects. Moreover, since they are highly biocompatible, there is little concern about the consequence of their ADME (absorption, distribution, metabolism, and excretion),^7^ which is usually an important safety issue when artificial nanoparticles (NPs) like polymers are used for the same aim.^8^ Therefore, exosomes are also regarded as intriguing natural drug delivery carriers.^9^, ^10^, ^11^, ^12^

The studies of exosomes confront technique difficulties. Analysis techniques with high sensitivity and specificity are currently lacking, thus in order to achieve quantitative analysis for clinic applications, a large pool of purified exosomes is usually required. However, the exosome purification technologies available now are far from satisfactory. Some studies showed that the methods used for exosome purification affect their heterogeneity, raising the concern about the fidelity of the purified exosomes to reflect the in vivo natural status.^13^, ^14^, ^15^ Moreover, some widely used methodologies based on the recognition of surface markers are recently found to as well enrich other multiple vesicle bodies like ectosomes.^16^ Therefore, more efficient and exosome‐specific purification and highly sensitive detection methods are eagerly required. An intriguing clinical application of exosomes is for drug delivery.^17^ For this aim, adequate exosomes must be produced from source cells and appropriate surface modifications are usually desired.^18^ Till today, approaches used for realizing these aims are unmature and have much room for improvement.

Nanotechnology has been widely explored in different fields of biomedicine in recent decades.^19^, ^20^ Properties of varieties of NPs, like high specific surface area,^21^ unique optical effects,^22^ and superparamagnetism,^23^ make them highly promising to revolutionize exosome studies. On one hand, nanotechnology provides novel strategies for exosome biogenesis regulation, purification, and analysis, making more sensitive and accurate functional readings possible.^24^ On the other hand, it helps to broaden the scope of exosome applications in biomedicine, which is impractical by traditional technologies.^25^, ^26^ Some significant progresses have been made in these fields in recent years. In this article, we will summarize the recent progress in NPs' applications for exosome biogenesis control, purification, quantitative detection, and manipulation for medical aims.

## 1 NPS FOR EXOSOME BIOGENESIS REGULATION

Recent studies indicated that exosomes play critical roles in regulation of normal physiological processes like pregnancy,^27^, ^28^ development and immune response,^29^ as well as in pathogenesis^30^ and disease progression.^31^, ^32^ Exosomes are also found to be associated with diseases like neurodegenerative and cardiovascular diseases and tumors.^5^ Because of this, modification of exosome biogenesis appears as a way of maintaining healthy physiology and preventing disease progression. The accumulated knowledge about the exosome biogenesis opens novel ways to manipulate major steps and factors in this process.^1^, ^33^, ^34^, ^35^

NPs have been reported to modulate exosome attributes. In one study, gold NPs (AuNPs) of 5 nm stimulated mouse embryonic stem cells to secrete exosomes which are distinct from the natural ones since they have higher rigidity and different protein expression profile.^36^ Moreover, unlike those produced in nontreated cells, these exosomes did not promote tumor cell migration in breast tumor cell model 4 T1, probably because they did not stimulate the expression or phosphorylation of metastasis‐promoting proteins like cofilin and extracellular regulated protein kinases (ERKs).^36^ This study suggests the possibility of employing NPs to host cells to produce safer exosomes as drug delivery carriers.

One widely used approach for exosome biogenesis regulation is to manipulate protein expression of important regulator genes involved in the process of exosome generation and conveyance.^37^ To do this, silencing molecules like siRNAs or antisense oligonucleotides or exogenous DNAs are usually applied.^38^ However, delivery of these molecules confronted low efficiency due to the lack of appropriate carriers. NPs have a high drug loading capability and are promising to break this limit. GTPase family proteins play important roles in multiple steps of vesicle trafficking including budding, docking, and fusion, thus are important regulators of exosome secretion.^39^ By carrying antisense oligonucleotides targeting a GTPase family member RAB27A, AuNPs significantly lessened exosome release from MCF‐7 and MDA‐MB‐453 cells.^40^ And the functionalized AuNPs can be carried over to the recipient cells by the secreted exosomes and continue to execute gene silencing. These pilot studies highlight the possibility of using NPs to regulate exosome biogenesis aiming to limit tumor metastasis.

Naturally, cells produce a small number of exosomes, which forms the major limitation of their therapeutic applications. In order to enhance the production, some mechanical methods, like iterative physical extrusion or freeze/thaw cycle have been reported.^41^, ^42^ A disadvantage of these methods is that the integrity of exosome membrane may be damaged. NPs have been enforced to stimulate higher exosome yield in cells. In one study, platinum NPs treatment induced six folds higher exosome production in cancer cells via an oxidative stress‐mediated mechanism.^43^ In another study, porous silicon NPs promoted autophagosome formation in human hepatocarcinoma cell line when used as a drug delivery carrier and stimulated 34 times more exosomes release.^44^ Positively charged iron oxide NPs entered mesenchymal stem cells via a dynamin‐dependent endocytosis mechanism, and in cells, they enhanced exosome secretion by stimulating the expression of factors involved in autophagosome and autolysosome formation including Beclin‐1 and Rab7.^45^ These studies showed that application of NPs is an easy and highly efficient way to improve exosome production.

Although the above success, when NPs were applied to cells, the increased exosome yield is often accompanied by cellular toxicity mediated by induction of oxidative stress and autophagy.^43^, ^44^ This cellular toxicity consequently decreased source cell viability and the alteration of exosome attributes, as well as leave stress markers in exosome cargos which may affect recipient cell physiology when exosomes are used as theragnostic delivery carriers.^45^ So in future, one of the study aims is to develop efficient NPs to stimulate exosome release but with minimal side effects to exosome‐derived cells.

## 2 NPS FOR EXOSOME PURIFICATION

Based on the difference in physiochemical and biological properties between exosomes and other components in the biological fluids, exosomes are traditionally purified by approaches including ultracentrifugation, ultrafiltration, precipitation, size‐exclusion chromatography, or immunoaffinity capture. These approaches have their own advantages and disadvantages **(**Table 1 **)**. The applications of NPs for exosome purification are mostly used in immunoaffinity capture. In this use, antibodies or aptamers targeting exosome surface molecules (mainly proteins or lipids) are conjugated to NPs. There are two attractive advantages when NPs are used for this purpose. First, because of the high specific surface area, a large amount of targeting molecules can be conjugated, thus enhance the binding affinity with exosomes. Second, varieties of NPs with different functions can be combined to form a multiply functionalized nano‐platform for realization of simultaneous isolation and quantification. Because of these advantages, exosomes can be purified under a mild condition in a more efficient manner. For example, by applying magnetic NPs, folate functionalized exosomes can be isolated with a high specificity in less than 2 h, compared to at least 4 h by applying ultracentrifugation.^52^ Another example is that Fe_3_O_4_@TiO_2_ NPs were used for exosome purification taking advantage of the binding between phospholipids of exosomes and TiO_2_ shell. By applying an exogenous magnetic field, 96.5% of exosomes can be purified within 5 min, an efficiency higher than any reported traditional approaches. And after simple wash step, the high enriched exosomes can be used for the following‐up quantification step.^53^ By using antibody‐conjugated magnetic nanowires, exosomes were isolated in less than 1 h and the purification yield reached more than 10 times than that obtained by ultracentrifugation (~150 × 10^8^ vs. ~10 × 10^8^ exosomes/ml).^54^

**FIGURE 1:**
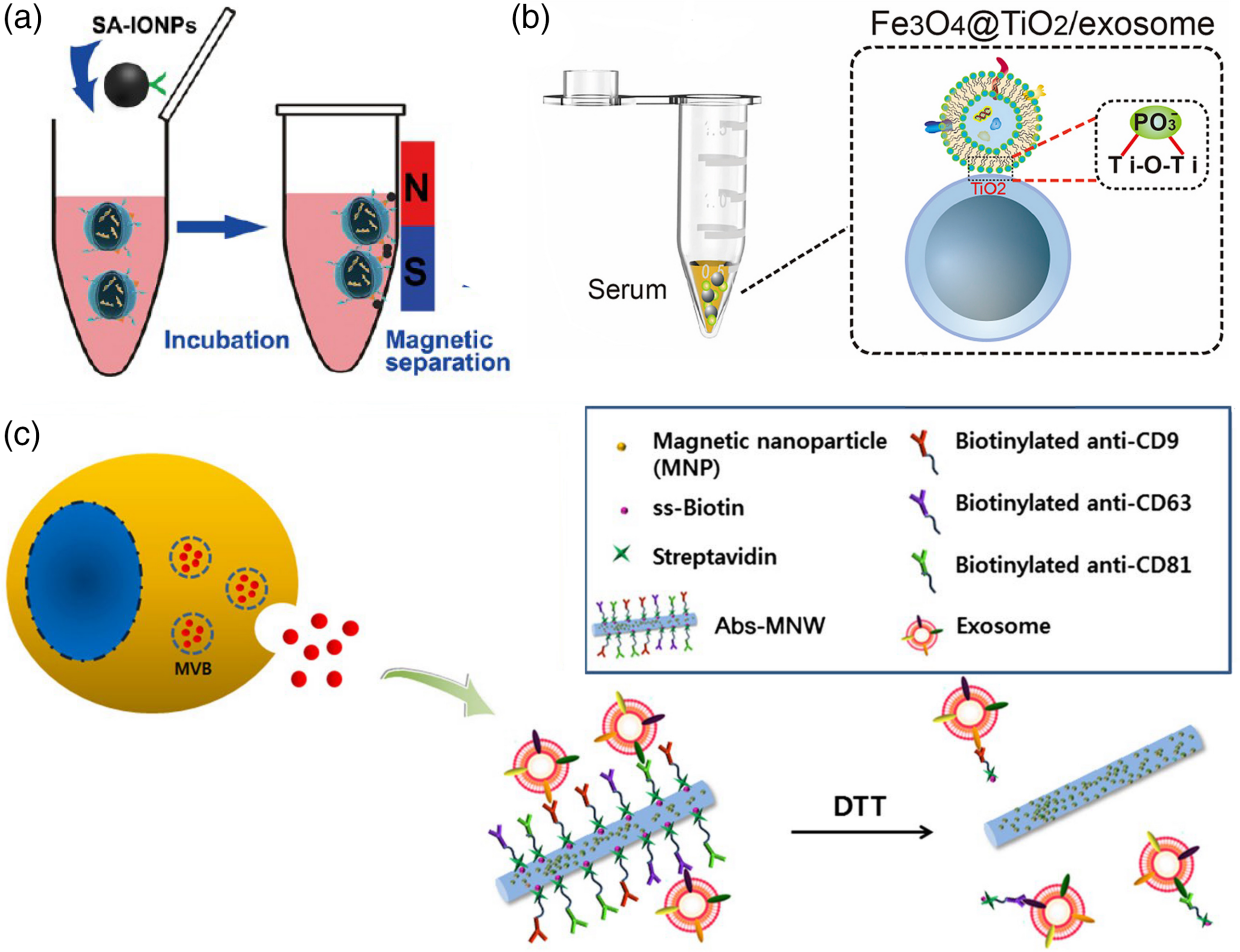
Mechanism of magnetic or TiO_2_‐based exosome isolation. (a) Schematic illustration of the isolation of streptavidin‐modified iron oxide nanoparticles (SA‐IONPs)/exosomes. By conjugation with SA‐IONPs, cell‐derived exosomes can be efficiently isolated from the supernatant using magnetic activated sorting (Image reprinted with permission from Reference [52]. (b) Mechanism of exosomes captured by Fe_3_O_4_@TiO_2_. Enrichment of exosomes is achieved via the bidentate binding between the phosphate groups on the surface of exosome and TiO_2_ (Image reprinted with permission from Reference [53]. (c) An illustration showing the antibody cocktail‐conjugated magnetic nanowires used for the isolation of circulating exosomes (image reprinted with permission from Reference [54])

The most widely used property of NPs is their superparamagnetism produced by magnetic NPs (Figure 1a).^52^ The targeting moieties can be any molecules natural to cells, like transferrin receptors from blood cells,^55^ or those designed to be carried by exosomes by a donor cell‐assisted membrane modification strategy,^52^ such as biotin (streptavidin as ligand) or folic acid (folic acid receptor as ligand).

**TABLE 1:** The advantages and disadvantages of protocols for exosome purification

Separation method	Principle	Advantages	Disadvantages	Reported yield	References
Ultracentrifugation	Speed related to the particle size/density	High purity, large volumes	Special equipment required; tedious process， time‐consuming (3–18 h)	275 μg/mg	[46, 47]
Immunoaffinity Capture	Specific binding between immobilized antibody and antigens on exosomes	High specificity/ purity and sensitivity; fast (1.5 h)	High cost; small volume (0.5 ml)	117 μg/mg	[47]
Size‐exclusion chromatography	Exosome size determines retention time	Simple operation; high purity; retention of biological activity; fast (0.5 h)	Dilution of sample required	1.0 × 10^12^/ml	[48]
Ultrafiltration	Membrane intercept	Simple operation; large volume; fast (0.5 h)	Low purity, membrane damage	2 × 10^10^ /10^6^ cells	[49]
Polymer‐based Precipitation	Exosome solubility/dispersion changed by polymers	Simple operation; large sample volume, 0.5 –12 h	Lower activity of exosome, low purify	4.5 × 10^10^/ml	[50]
Microfluid‐based techniques	Fluid channels are designed according to the characteristics of exosomes	High sensitivity and low cost, high purify; fast (0.5 h)	Difficultly in handling large sample	NA	[51]

When used for exosome purification, magnetic NPs can be directly modified on their surface by targeting molecules. For example, transferrin molecules were covalently conjugated to Fe_3_O_4_ NPs surface and used to isolate transferrin receptor positive exosomes in a magnetic field.^55^ Magnetic NPs can also be further modified using different types of NPs to form a multiply functionalized platform. For example, Fe_3_O_4_ NPs can be coated by TiO_2_ NPs to form a composite with core‐shell structures, taking advantage of strong binding affinity between TiO_2_ with hydrophilic phosphoric acid of exosomes. This composite can be used to realize aptamer‐free exosome isolation (Figure 1b).^53^ Fe_3_O_4_@Gold core‐shell NPs were also developed. In this composite, gold shell surface was conjugated with aptamers to specifically recognize exosome surface markers, and also serves as a SERS test tag.^56^ Moreover, detection tags, like AuNPs for surface‐enhanced Raman scattering (SERS) immunoassay, can be added to NPs surface for in situ quantification without the requirement of elution, a critical step to lose the isolated samples. Magnetic nanowires doped inside with magnetic nanospheres were used to efficiently catch circulating exosomes (Figure 1c).^54^ The thermal responsibility of magnetic NPs was also used for exosome purification.^57^

NPs may also aid exosome purification by other mechanisms. Recent studies find that nanometer scale topography can be used for tumor cells or cell−/sub‐cell size organisms (like bacteria) capture based on cell‐nanostructure interaction.^58^ This emerges as an important mechanism underlying microfluidic chip technology.^59^, ^60^, ^61^ By using manufacturable silicon processes, an array with uniform nanoscale gap sizes can be produced, which has been reported to efficiently separate exosomes.^62^ NPs like graphene oxide are good candidates to be used as coating materials to make nano‐interfaces with enhanced capture area while suppressing nonspecific adsorption.^63^, ^64^

## 3 NPS FOR QUANTITATIVE EXOSOMES DETECTION

Exosomes are valuable liquid biopsy biomarkers for clinical uses such as noninvasive early cancer diagnosis and evaluation of therapeutic efficacy.^65^, ^66^, ^67^, ^68^ By qualitatively or quantitatively analyzing disease‐related biomarkers of exosomes, the disease status can be detected. Without the engagement of nanotechnology, exosome detections relied on some traditional methods including western blot,^69^ enzyme‐linked immunosorbent assay,^70^ and enzyme‐assisted or enzyme‐free amplification‐dependent fluorescence assays.^71^, ^72^ The shortcomings of these methods are either low sensitivity, low specificity, or time‐consuming. For example, from one study, exosomes in supernatant from culture of at least 3.5–10 million cells were required for western blot analysis.^69^ The studies of using NPs for exosomes‐based clinic use aim to develop fast, cheap, and highly sensitive techniques for diagnosis of diseases like cancers, cardiovascular diseases, neurodegeneration, and metabolic diseases. Some laboratories develop different strategies to reach this aim and their works using real clinic samples show promises to transplant these strategies for clinical application (Table 2).

**TABLE 2:** Examples of NPs‐based exosome detection

Type		Purification method	Substrate	Signal detection	Detection limit	References
Fluorescence	Solution‐based	Immunoaffinity capture	Cholesterol‐modified magnetic beads	CuO NPs	48 k/μl	[73]
	Microfluidic‐based	Immunoaffinity capture	Graphene oxide/polydopamine nano‐interface	FITC‐labeled CD81	1 k/μl	[63]
	Paper‐based	Ultracentrifugate	Paper‐supported aptasensor	Gold nanorod modified with DNA	1.1 k/μl	[74]
Electrochemistry	Conventional electrode based	Magnetic bead separation	Glassy carbon	Dissolved CdSe quantum dots	100/μl	[75]
	Screen‐printed electrode‐based	Simple centrifugation	Nanostructured gold	AgNPs or CuNPs	50/sensor	[76]
	Micropatterned electrode‐based	Ultrafiltration	Gold NPs	Methylene Blue	1 million /ml	[77]
Colorimetry	Paper‐based lateral flow	Ultracentrifugation	Nitrocellulose membrane	Anti‐CD63 labeled with AuNPs	0.8 million/ml	[78]
	Solution‐based	Magnetic bead	Magnetic bead‐labeled CD63 aptamer	Ag@Au	160/μl	[79]
Resonance	μNMR	Ultracentrifugation	Magnetic NPs	N/A	10^4^/ml	[80]
	SPR	Microfluidic device	Nanoporous gold nanocluster	Antibody AuNPs	1000/ml	[81]
	LSPR	Ultracentrifuge	Gold nanoislands	N/A	0.194 μg/ml	[82]
	SERS	Immunoaffinity capture	Glass solid	Ag@Au	540/ml	[83]
		Magnetic bead	Magnetic substrate	AuNPs@SERS reporter	32–200/ml	[56]

Abbreviations: AuNPs, gold nanospheres; CuNPs, coppernanospheres; FITC, fluorescein isothiocyanate; LRSPP, long‐range surface plasmon polariton; LSPR, local surface plasmon resonance; SERS, surface‐enhanced Raman scattering nanorods; SPR, surface plasmon resonance.

Different strategies for quantitative analysis of exosomes have been developed. When used for quantitatively testing exosomes in liquid biopsy, some surface markers on exosomes are usually targeted.^84^ In this strategy, the targeted surface markers in exosomes can be classified into two categories. The first one belongs to the group of intrinsic molecules. These molecules include CD63, Alix, and HSP70 which are common to varieties of exosomes and are usually used to identify the isolated crude products.^85^ Another group is composed of specific proteins closely correlated with the occurrence or development of diseases, such as protein PD‐L1 in melanoma cell‐derived exosomes and EpCAM in exosomes from epithelial malignant tumors like lung and colon cancers.^86^, ^87^, ^88^ These markers are valuable for clinical diagnosis. To target the disease‐correlated markers, either their respective antibodies or oligo DNA named aptamers are used.^89^, ^90^ Compared to antibodies, the advantages of aptamer applications, like lower cost and ease of modifications, are attracting more interests in recent years.^91^ To quantitatively monitor in vivo distribution, exosomes can also be labeled by internalizing contrast agents. An example is that AuNPs were in vitro encapsulated inside exosomes. After administration into animals, both distribution and accumulation quantity in tissues can be monitored by in vivo CT imaging.^92^ Some other information molecules like microRNAs that reside in exosome compartment are also reported as target for quantitative detection aim.^24^

### 3.1 Fluorescence‐based detection

Because of their excellent properties like size‐regulable emission spectra and stronger photoluminescence performance compared to conventional organic fluorophores, quantum dots (QDs) are ideal fluorophores for fluorescent detection of exosomes. In one study, cadmium selenide (CdSe) QDs conjugated with IL13 bound exosomes released from glioma stem cells which express receptor IL13Rα2.^93^ Copper ions dissolved from CuO NPs can form copper NPs (CuNPs) under reduction condition, and the latter emit fluorescence in an intensity proportional to the concentration of copper ions. This mechanism was reported for exosome quantification.^73^

**FIGURE 2:**
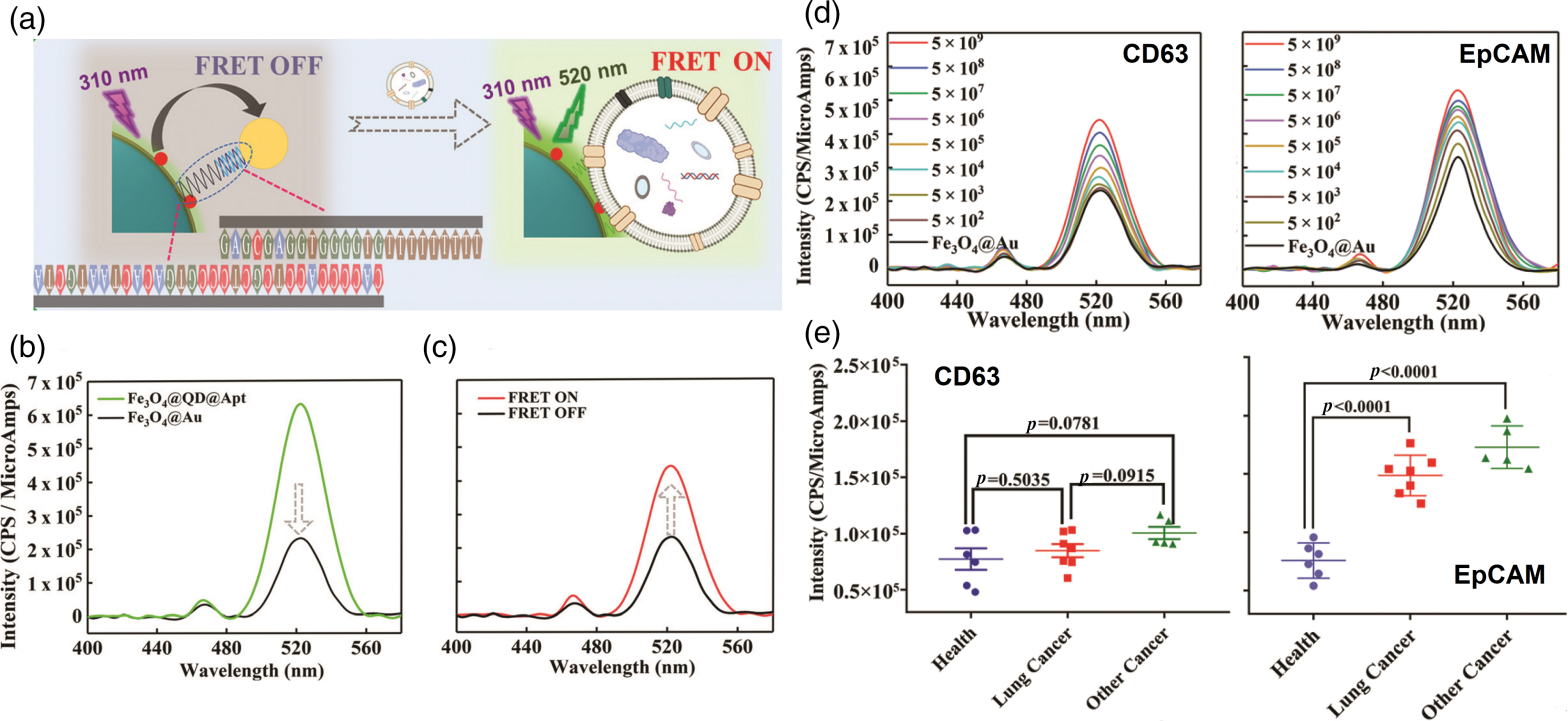
Exosome detection based on Förster resonance energy transfer (FRET) by the fluorophore‐quencher pair of quantum dots (QDs) and AuNPs. (a) Schematic illustration of a FRET‐based exosome detection strategy. (b) The fluorescence of QD on Fe_3_O_4_ nanoparticles (NPs) (Fe_3_O_4_@QD@Apt, green line) is quenched by the formation of complex directed by a complementary DNA conjugated on AuNPs (Fe_3_O_4_@Au, black line). (c) Fluorescence intensity increase based on FRET ON. (d) The fluorescence intensity recovery occurs in AptCD63‐ and AptEpCAM‐containing systems with the addition of exosomes. (e) Detection of the CD63 and EpCAM expression on serum‐derived exosomes using Fe_3_O_4_@Au systems functionalized with AptCD63 or AptEpCAM (image reprinted with permission from Reference [94])

QDs are also used for quantitative exosome analysis based on a mechanism known as Förster resonance energy transfer (FRET). In one study, the fluorescence of QDs on the surface of Fe_3_O_4_ NPs was designed to be quenched by AuNPs, which are drawn to proximity by pairing of aptamers conjugated to them (Figure 2). When incubated with exosomes, competitive binding of exosome surface markers with the aptamers expelled AuNPs, resulting in an exosome concentration‐dependent restoration of QDs fluorescence. When lung cancer epithelial marker EpCAM was targeted by aptamer, this system can detect as low as 13 exosome particles/ml sample with 100% accuracy, showing great clinical application potential.^94^ In this strategy, three different NPs were used for different applications. Fe_3_O_4_ NPs were used as both a platform carrier and exosome isolation tool, while QDs and AuNPs formed a fluorophore‐quencher pair.

To realize these applications, exosomes were labeled with QDs by different approaches. Like other types of extracellular vesicles, exosome surface is rich in primary amines, which has been used to engage amine‐reactive NHS‐ester reaction for QDs labeling.^95^ Antibodies and aptamers were also utilized to conjugate QDs to exosome surface.^96^, ^97^, ^98^

Two‐dimensional NPs have a structure of single atomic layer and incline to form π‐π electron stacking interactions with biological and chemical molecules like DNA, proteins and fluorescent molecules, thus possessing a high distance‐dependent fluorescence quenching capability.^99^, ^100^ In recent years, different types of 2D NPs have raised great interests for their potential applications as a nanoquencher in FRET‐based biosensor platform. By using graphene oxide, EpCAM‐positive exosomes derived from colorectal cancer cells were quantitatively determined in a fast manner (~30 min) although in a medium sensitivity (2.1 × 10^4^ particles/μl).^101^ By using Ti_3_C_2_ MXenes nanosheet, PSMA (prostate‐specific membrane antigen) and TPK‐7 (a marker of several cancer types) positive exosomes were measured in a sensitivity 1000 times higher than the traditional ELISA method.^102^

Some NPs are also used in a similar technology named luminescence resonance energy transfer (LRET) for exosome quantification.^74^ In this strategy, upconversion and AuNPs are used and a sensitivity of 1000 particles/μl was reached by using CD63 as a test aptamer.

### 3.2 Electrochemistry‐based detection

Oxidation potentials and degradability of metal NPs make them useful in electrochemical quantification of exosomes. When the oxidation potentials of two metal NPs are sufficiently differentiated, multiple markers on exosome surface can be detected simultaneously. For example, in order to simultaneously test PSA and EpCAM level in serum exosomes derived from prostate cancer patients, silver NPs (AgNPs) and CuNPs are used because their oxidation potentials fall in the potential window of gold electrodes and are well separated. When a linear sweep voltammetry is applied, they are oxidized under different voltages in an intensity proportional to the quantity of their surface markers.^76^ CdSe QDs functionalized with the HER‐2 and FAM134B antibodies, which target respective exosome markers of breast and colon cancer cells, bind with exosomes in a proportion to their amount. The bound CdSe QDs will dissociate Cd^2+^ under acidic condition which can be quantified by anodic stripping voltammetry.^75^ Besides, DNA nanotetrahedron‐modified electrode employed aptamer to capture more exosomes and, therefore, enhanced the detection sensitivity.^103^

### 3.3 Colorimetry**‐**based detection

Two intrinsic properties of NPs make them applicable in colorimetry‐based exosome quantification. First, some NPs, like AuNPs, show different colors dependent on their aggregation state. Second, NPs with peroxidase activity can catalyze colorimetric reactions.^104^, ^105^, ^106^ AuNPs coated by aptamers are well dispersed in high salt solution. When exosomes are added, the complex of aptamer‐AuNPs are replaced by aptamer‐exosome, resulting in AuNP aggregation and a red to blue color change, which can be monitored by absorption spectroscopy.^107^ By this mechanism, both ubiquitous and exosome‐specific markers can be accurately tested in a time scale of minutes.

Binding of other molecules can change the intrinsic enzymatic activity of NPs, a property which has been exploited for exosome quantification. For example, graphitic carbon nitride nanosheets bound with single‐strand DNA (aptamers) shows a four times higher peroxidase activity compared to naked ones, and the catalytic activity is proportionally decayed when exosomes carrying the respective marker molecules competitively bind and strip the aptamers from the nanosheets. The change in the catalytic activity can be quantified by colorimetric measurement of H_2_O_2_‐mediated TMB oxidation.^105^

### 3.4 R**esonance‐based detection**

**FIGURE 3:**
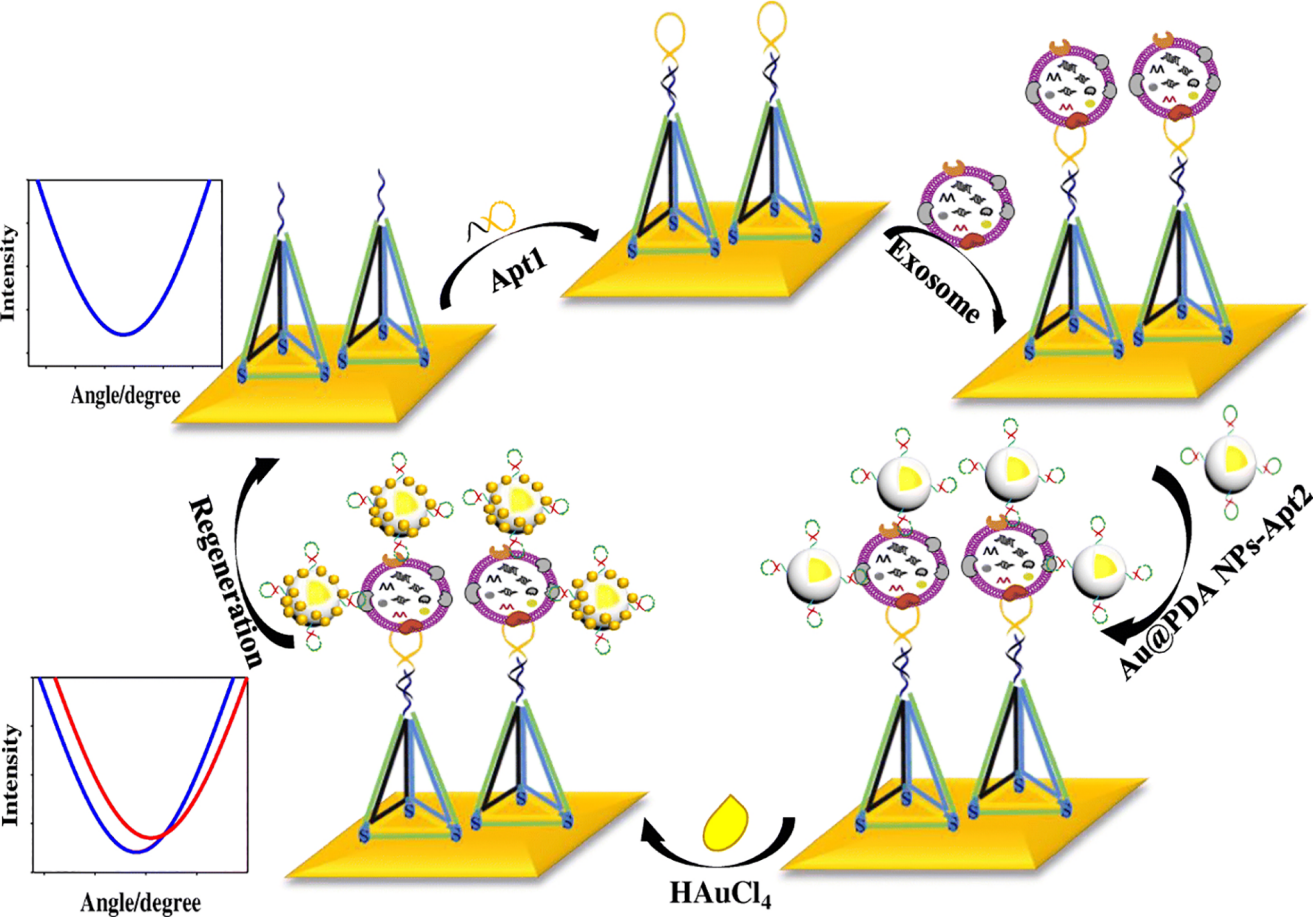
A surface plasmon resonance (SPR) strategy for exosome detection based on a three‐step process. In step 1, aptamer 1 is linked to a DNA tetrahedron probes (DTPs), which are supported on an Au film to prevent gold deposition on the surface. In step 2, exosomes are captured by the aptamer 1 which is complementary to DTPs, and aptamer 2 (here it is CD63)—linked Au@PDA NPs recognize and bind exosomes. In step 3, HAuCl_4_ is introduced and in situ reduced to AuNPs by polydopamine coated on the AuNP surface. The reduction results in a further enhanced SPR signal (image reprinted with permission from Reference [110])

Optical techniques based on light‐matter interactions have been developed for extracellular vesicles including exosomes analysis. Compared to the electrochemical methods, optical detection has higher reproducibility. These optical techniques include surface plasmon resonance (SPR), local surface plasmon resonance (LSPR), long‐range surface plasmon polariton (LRSPP), and surface‐enhanced Raman scattering (SERS). AuNPs, including gold nanospheres and nanorods (AuNRs) are the most used NPs because they resonantly couple with the surface plasmas of the biosensor chip, thus acting as a signal amplifier.^81^, ^82^ Different groups have reported their studies using this strategy to analyze exosomes derived from lung cancer and hepatic carcinoma patients.^81^, ^108^, ^109^ According to these studies, a limit of detection as low as 500 exosomes/μl sample can be achieved, which is much lower compared to that obtained by other protocols like colorimetry‐ and fluorescence‐based ones. This mechanism was also applied for in vivo exosome imaging.^92^ The higher sensitivity of AuNPs‐based plasmon biosensor attributes to the fact that a high density of local AuNPs on exosomes can be achieved by different methods. First, AuNPs conjugated with different antibodies/aptamers can be used to recognize different surface ligands simultaneously. Second, antibodies/aptamers conjugated AuNPs can be further modified by reductants (e.g., polydopamine) in order to in situ produce AuNPs from HAuCl_4_, which will further amplify plasmon resonance signals (Figure 3).^110^

## 4 EXOSOMES FOR NPS‐BASED DISEASE THERAPY

The requirements for an ideal chemotherapy strategy, such as targeted drug delivery and high therapeutic efficacy, inspire people to hybrid NPs and exosomes in a single system. In this hybrid, exosomes usually function as natural nanocarriers with incomparable advantages like higher biocompatibility, extended blood half‐life, and capability of translocation through physical barriers and target‐homing.^1^ Meanwhile, NPs per se may act as a therapeutic reagent or function as drug deliverers. The complementary properties of exosomes and NPs can be well taken advantages of in this system. Exosomes as natural drug delivery carriers have limitations like low payload capability, which is often blamed to be the reason of low therapeutic efficiency after in vivo administration.^111^ This limitation can be overcome by NPs, which can load significant amount of drugs, protect them from degradation, and realize controllable drug release by proper chemical modifications.^112^ Although the targeting capability of NPs can be entitled by conjugation of targeting ligands on NP surface, it is not uncommon to find that the targeting ligands deteriorate the stability and change in vivo distribution of NPs after administration by a mechanism involving corona formation on a NP surface.^112^ The use of exosomes as targeting moieties will help avoid such problems. The NPs‐exosome hybrids showed considerably increased therapeutic efficiency in different biomedical applications (Table 3).

**TABLE 3:** Examples of NPs‐exosome hybrid for biomedical applications

NPs	Exosome‐origins	Engineering methods	Cell/animal models	Applications	Efficiency	References
PEGylated spherical HGNs, 45 nm	B16‐F10 cells	HGNs stimulated exosome release by incubation with cells	B16‐F10 cells	NIR‐based cancer hyperthermia	Two times higher temperature than that in control after 30 min	[37]
PMA‐coated Au‐BSA@Ce6 NPs, 6–9 nm	Gastric cancer patients' urine	Exos‐PMA/Au‐BSA@Ce6 by electroporation with NPs and exosomes	MGC‐803 (a human gastric cancer cell line) tumor‐bearing mice	Cancer‐targeted photodynamic therapy	Deeper permeability and superior retention than exosome absence group; 20 days life span extension than PBS group.	[113]
Black phosphorus QDs, 1–3 nm	Serum from hyperthermia‐treated mice	Sonication of NPs/exosomes mixture	LLC/RAW264.7/ 4 T1 cells; Balb/c nude mice bearing LLC‐induced lung cancer	Anticancer photo‐nanovaccine	Long‐term PTT; higher tumor temperature and targeting efficacy; higher immune system activation efficiency.	[114]
TAT modified 2D vanadium carbide (V_2_C) QDs, nanosheet, 16 × 2.5 nm	MCF‐7 cells	Exos‐RGD by cell culture; V_2_C ‐TAT@Ex‐RGD by electroporation	MCF‐7/NHDF/A549 cells; MCF‐7 tumor‐bearing BALB/c nude mice	Nucleus‐targeting low‐temperature photothermal therapy and fluorescence imaging, PAI and MRI multi‐model tumor imaging	Higher photothermal conversion efficiency than other nano‐PTAs; 2.84 times stronger nucleus targeting ability than V2C‐TAT; 3.73‐fold higher MR signal than control; higher tumor accumulation and negligible side effects	[115]
MIL‐88A, an iron‐based MOFs, roundish, 52 nm	Hela cells	Exos‐MIL‐88A by lipid fusion with NPs and exosomes	Hela cells	Drug delivery	3 times higher IC50 compared with free SBHA	[116]
Spheric SPIONs	Raw264.7 cells	RGE‐Exo‐SPION/Cur formed by electroporation and click chemistry	Bel‐7404/ U251 cells; tumor‐bearing mice	Imaging and therapy of glioma	Longer survival than control groups	[117]
Dox loaded spheric PSiNPs with11 nm pore	Bel7402 cells	DOX@Exos‐PSiNPs by NPs incubation with cells	H22 CSCs tumor spheroids; H22 tumor‐bearing mice	Drug delivery	2 times targeted in vivo and in vitro tumor accumulation; 3.2 times DOX retention; higher tumor growth inhibition; 40 days longer survival time	[44]
Pd nanosheet, 1.4 nm in thickness	A549 cells	In situ synthesis of Pd‐Exos from K_2_PdCl_4_	A549/U87/ RAW246.7 cells	Anticancer prodrug delivery	Targeted cancer therapy	[118]

Abbreviations: BSA, bovine serum albumin; Cur, curcumin; CSC, cancer stem cells; Dox, doxorubicin; HGNs, hollow gold nanoparticles; exos: exosomes; NIR, near‐infrared; NP, nanoparticle; PMA, amphiphilic polymer; PSiNPs, porous silicon nanoparticles; PTT, photothermal therapy; QDs, quantum dots; RGE: RGERPPR peptide; SBHA, suberohydroxamic acid; SPIONs, superparamagnetic iron oxide nanoparticles; TAT, TAT peptide (for nucleus targeting).

Two different approaches have been reported to prepare NPs‐exosome hybrids. In the first approach, NPs are added to the cell culture to stimulate cellular endocytosis, by which NPs are loaded inside exosomes. In the second approach, NPs are encapsulated into the isolated exosomes in tubes by various physical methods like electroporation or freeze–thaw cycles, or by in situ synthesis of NPs starting from ions. A reported in situ synthesis example is the delivery of catalyst Pd nanocrystals starting from Pd ions.^119^

### 4.1 In tumor therapy

**FIGURE 4:**
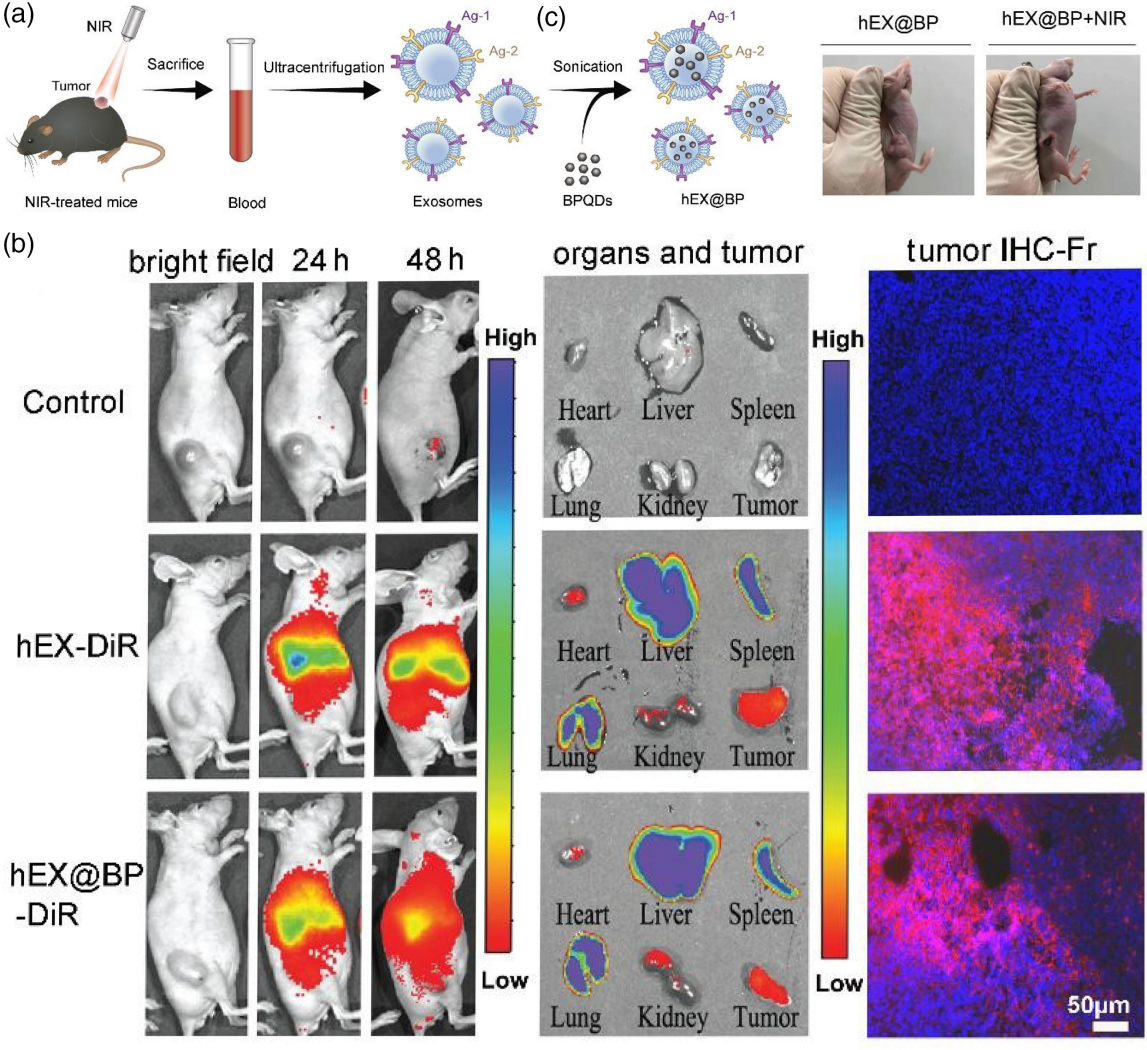
In vivo photothermal performance of a hybrid of black phosphorus quantum dots (BPQDs) encapsulated in exosomes (hEX@BP) in lung cancer mouse models. (a) Graphic illustration of hybrid construction. Exosomes (hEX) are extracted from the plasma of hyperthermia‐treated tumor‐bearing mice, and are used to encapsulate BPQDs by an ultrasonic method. (b) Left: Live imaging of mice with a lung xenograft tumor injected with DiR‐labeled hEX and hEX@BP. Middle: Ex vivo images of lung tumor and whole‐body organs (heart, liver, spleen, lung, and kidney). Right: Fluorescent frozen section of tumors (bar: 50 μm) from different‐treatment mouse groups. hEXs are stained with DiR (red) and DNA are stained with DAPI (blue). (c) Smaller tumor size in NIR radiation treated mice (Image reprinted with permission from Reference [114])

When used in exosome‐mediated tumor therapy, NPs are playing different roles in the NPs‐exosome hybrids. First, they per se may act as therapeutic reagents. Hollow AuNPs can significantly convert near‐infrared light into heat and thus are often used in cancer photothermal therapy.^120^ When they were encapsulated into exosomes by an endogenous method and were carried into melanoma cells, they efficiently increased the temperature of cells in culture under near‐infrared laser irritation.^37^ This photothermal effect was also realized by hollow AuNPs delivered by exosomes in mesenchymal stem cells.^121^ Noticeably, because of their homing selection, exosomes derived from mesenchymal stem cells only enter mesenchymal cells but no other cell types in culture. Another type of NPs, black phosphorus (BP) nanosheets were also reported in an exosome‐mediated tumor photothermal therapy. In this study, after intravenous injection into mice, BP carrying exosomes were accumulated in tumors because of their homing selection,^122^ and application of near‐infrared laser irritation efficiently produced local heat and ablated tumors (Figure 4).^114^

Second, NPs work as a drug deliverer. In one study, Dox/siNPs‐encapsulated exosomes were successfully produced by a pre‐loading approach, in which doxorubicin loading porous silicon NPs were exposed to cultured hepatocarcinoma cells and they were released by cells in the form of Dox/siNP‐loaded exosomes. When administrated by intravenous injection to mice bearing hepatocarcinoma tumors, the exosomes accumulated in a high efficiency in tumors and doxorubicin were released from NPs killing both bulk tumor cells and cancer stem cells.^44^

The targeting capability of exosomes can be endowed by genetically engineering of parental cells. For example, by fusing neuron‐specific peptide gene to Lamp2b (a ubiquitous exosome membrane protein) in dendritic cells, a neuron‐targeting exosome pool was achieved.^123^ In another study, an Arg‐Gly‐Asp (RGD) peptide, which specifically recognizes the integrin α_v_β_3_ of tumor cells and vasculatures, was incubated with cancer cell line to generate RGD expressing exosomes (Exo‐RGD). QDs coated with TAT peptide (for nucleus targeting) were encapsulated into Exo‐RGD by electroporation. When applied to animal models, this theragnostic platform showed good biocompatibility and long circulation time, and targeted cancer cells with a high transfection efficacy.^124^

In BALB/c mice, the exposure to magnetic NPs generated exosomes in the alveolar region, which consequently stimulated T‐cell activation.^125^ This provides a mechanistic explanation for toxicity caused by NPs administration, but meanwhile, it also implicated the possibility of using this strategy for tumor nano‐vaccine development.^114^

### 4.2 In therapy for other diseases

**FIGURE 5:**
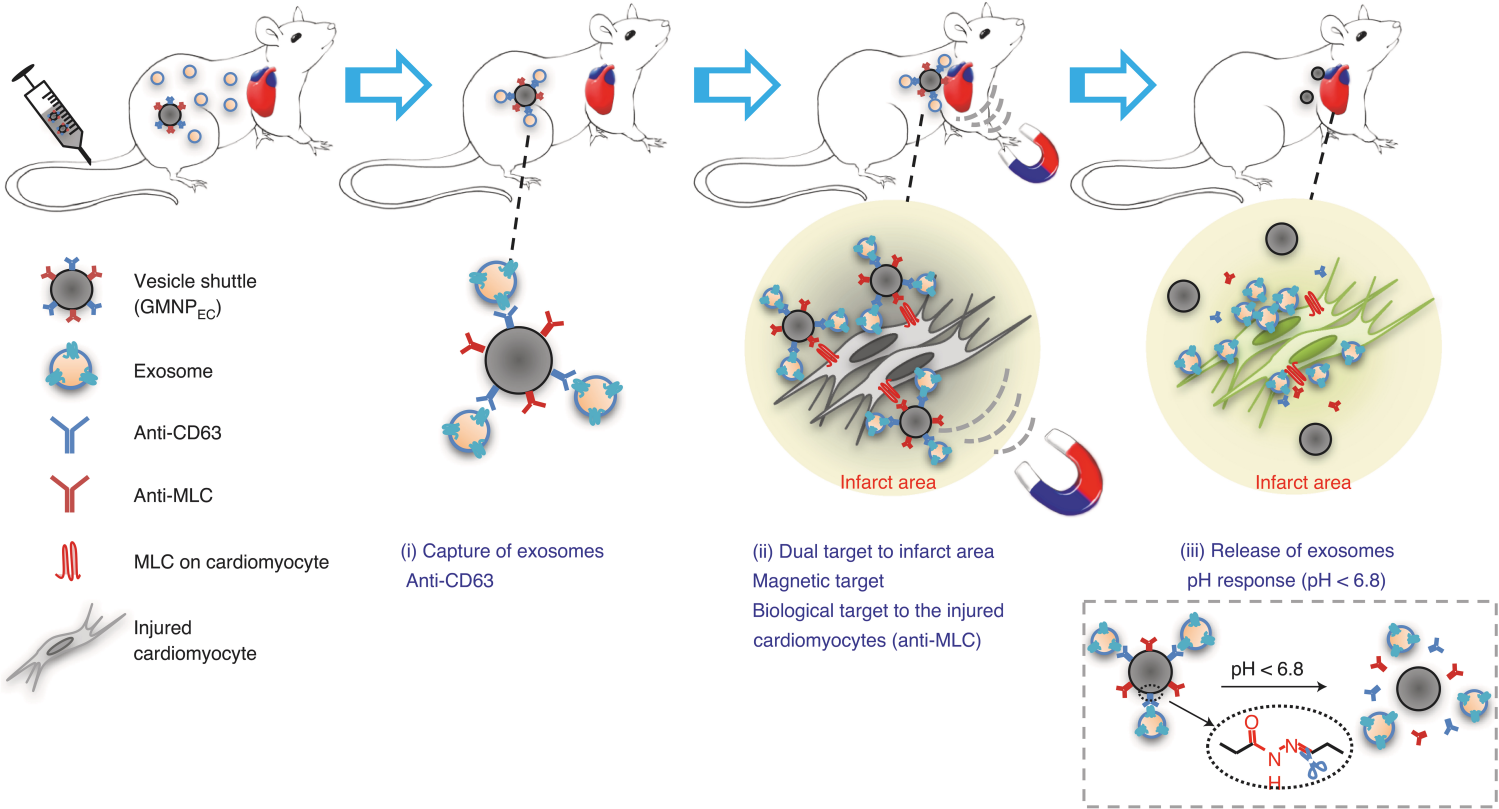
Treatment of infarcted heart tissue via the capture and local delivery of circulating exosomes through antibody‐conjugated magnetic nanoparticles (NPs). The magnetic NPs, designated as GMNP_EC_, consist of a Fe_3_O_4_ core and a silica shell that is decorated with poly (ethylene glycol) conjugated to two antibodies respectively recognizing CD63 and myosin‐light‐chain (MLC) via hydrazone bonds. While anti‐CD63 antibodies capture and attach to endogenous circulating exosomes, anti‐MLC antibodies lead exosomes to target MLC on the damaged cardiomyocytes when GMNP_EC_ are enriched in the infarct area by the application of a local magnetic field. In the infarct area, exosomes are released from GMNP_EC_ due to the acidosis‐induced cleavage of hydrazone bonds. In animal models of myocardial infarction, the accumulation of CD63‐expressing exosomes in infarcted tissue leads to reductions in infarct size as well as improves left‐ventricle ejection fraction and angiogenesis (Image reprinted with permission from Reference [126])

The combination of NPs and exosomes was also exploited for treatment of other diseases. In a notable study, core‐shell magnetic NPs were conjugated with dual antibodies, with one (CD63) against antigens on exosome surface and the other (myosin‐light‐chain, MLC) targeting recipient cells (the injured cardiomyocytes). These dual functionalized NPs were used as vesicle shuttle of capturing and delivering endogenous exosomes to the targeted area, where exosomes were selectively released from NPs to generate the therapeutic effect for myocardial infarction, including promoted angiogenesis, reduced infarct size, and improved left‐ventricle ejection fraction in animal model.^126^ Because the selection of targeted antigens on exosome surface can be further optimized, the specificity and efficiency to deliver exosomes still has room of improvement. Thus, this approach has great promises of clinic applications for myocardial infarction treatment. Studies have shown that exosomes containing pro‐coagulation and pro‐inflammatory factors contribute to thrombosis in myocardial infarction.^127^ This finding justifies a strategy for infarction treatment, in which exosomes containing these factors can be recognized and tethered by NPs with conjugation of appropriate antibodies, thus reducing their accumulation in the infarct site and ameliorating infarct size. The same strategy was also used for cerebral disease treatment^92^, ^128^ and monitoring and improving fetal health.^129^ In both applications, the capability of exosomes to breakdown physiological barriers (blood–brain barrier [BBB]^129^ and placenta barrier^130^) was taken use of. Drugs carried by this system can be more efficiently delivered to targeted sites to realize the maximal therapy efficacy.^131^, ^132^, ^133^ Moreover, therapeutic properties of NPs, like the photothermal effect, capability to activate immunity of targeted cells, and long retention time in tumors, can synergize drug therapy efficiency (Figure 5 **)**.^113^, ^126^, ^134^

## 5 CONCLUSIONS AND PERSPECTIVES

Nanotechnology is aiding exosome studies and applications in medicine in different ways. Besides improving purification efficiency and detection sensitivity of those current technologies, the use of various NPs as theragnostic carriers significantly enhance the drug loading capability of exosomes in disease therapy. Meanwhile, the homing selection property makes exosomes ideal targeting moieties in NPs‐based drug delivery system and help to overcome limitations caused by conjugation of targeting molecules on NPs' surface. Even though big progress has been achieved in recent years, till today there is no approval for commercial diagnostic kits or drugs based on NPs‐exosome hybrids. This is because some major obstacles confronting clinical applications of NPs‐exosome hybrids. For exosome detection in liquid biopsy, most of currently developed technologies are on the stage of proof‐of‐concept and successful detection is dependent on the purification of exosome. Although showing clinically satisfactory sensitivity and specificity, the steps before the detection, including operation of sampling, transportation, and purification, are yet to be standardized to ensure test repeatability, reproducibility, and minimal false negative ratio. Reliable and specific markers for tumor diagnosis and prognosis evaluation are still lacking. When used for in vivo applications like drug delivery, the limitations of the NPs‐exosome hybrids include the concerns raised by the potential toxicity of various artificial NPs. Moreover, by current approaches, it is still difficult to produce sufficient exosomes with constant quality and controllable properties like composition uniformity. The current endeavors of researchers are largely focusing on these issues and the accumulation of knowledge in NPs safety studies and exosome biology is promising to fill these gaps in future.

## CONFLICT OF INTEREST

The authors declare no conflict of interest.

## AUTHOR CONTRIBUTIONS

**Yabin Wang:** Writing – review and editing (equal). **Bing Yan:** Project administration (lead); writing – review and editing (supporting).

### PEER REVIEW

The peer review history for this article is available at https://publons.com/publon/10.1002/btm2.10269.

## Data Availability

There are no supplementary data available for this article.
